# Comparative analysis of ischemic and hemorrhagic stroke hospitalization rates in end-stage kidney disease and kidney transplant patients with and without atrial fibrillation

**DOI:** 10.1371/journal.pone.0310181

**Published:** 2024-12-16

**Authors:** Tyler Canova, Rochell Issa, Patrick Baxter, Alexander J. Didier, Alicia Nahhas, Meng-Hao Li, Ian Thomas, Naoru Koizumi, Ehab Eltahawy, Obi Ekwenna

**Affiliations:** 1 Department of Medicine, The University of Toledo College of Medicine, Toledo, Ohio, United States of America; 2 Department of Internal Medicine, Mayo Clinic, Rochester, MN, United States of America; 3 Department of Internal Medicine, Cleveland Clinic Foundation, Cleveland, Ohio, United States of America; 4 Schar School of Policy and Government, George Mason University, Arlington, Virginia, United States of America; 5 Department of Nephrology & Transplant, Mount St. John’s Medical Centre, St. John’s, Antigua & Barbuda; 6 Department of Cardiovascular Medicine, The University of Toledo Medical Center, Toledo, Ohio, United States of America; 7 Department of Urology and Transplantation, The University of Toledo Medical Center, Toledo, Ohio, United States of America; BSMMU: Bangabandhu Sheikh Mujib Medical University, BANGLADESH

## Abstract

**Introduction:**

Atrial fibrillation (AF) in end-stage kidney disease (ESKD) and kidney transplant (KTx) recipients presents challenges in stroke risk management. This study aimed to compare hospitalization rates for ischemic and hemorrhagic cerebrovascular events in ESKD and KTx patients with and without AF.

**Methods:**

Using the National Inpatient Sample (2005–2019), retrospective analysis was conducted on hospitalizations for ESKD and KTx patients with and without AF. Baseline characteristics and hospitalization rates for five cerebral ischemic conditions and one hemorrhagic condition were compared. Descriptive statistics and t-tests were employed for analysis.

**Results:**

Among ESKD patients, those with AF exhibited significantly higher hospitalization rates for ischemic stroke, including 1)Cerebral infarction due to thrombosis, embolism, occlusion (0.11% vs. 0.08%,p<0.001), 2)Cerebral infarction due to thrombosis, embolism, and unspecified occlusion (1.93% vs. 1.51%, p<0.001), 3)Artery occlusion resulting in cerebral ischemia (1.37% vs. 0.93%,p<0.001), 4)Cerebral artery occlusion resulting in cerebral ischemia (0.48% vs. 0.42%,p<0.001), while experiencing lower rates of intraoperative and postprocedural cerebrovascular infarction (0.88% vs. 0.97%,p<0.001) compared to those without AF. Conversely, KTx patients with AF showed increased hospitalizations for hemorrhagic stroke, particularly nontraumatic intracranial hemorrhage (0.79% vs. 0.56%,p<0.001), compared to those without AF. However, they did not exhibit significant differences in hospitalization rates for most ischemic conditions, except for cerebral infarction due to thrombosis, embolism, and unspecific occlusion (1.62% vs. 1.11%,p<0.001) and artery occlusion resulting in cerebral ischemia (0.84% vs. 0.52%,p<0.001).

**Conclusion:**

Our findings reveal patterns in hospitalization rates between ESKD and KTx patients with AF compared to those without AF, with ESKD patients with AF exhibiting higher rates of ischemic stroke compared to ESKD patients without AF and KTx patients with AF showing increased hospitalizations for hemorrhagic stroke compared to those without AF. These findings demonstrate the impact of AF on hospitalization rates for ischemic and hemorrhagic cerebrovascular events in both ESKD and KTx patients.

## Introduction

Atrial fibrillation (AF) is common in individuals with end-stage kidney disease (ESKD), affecting approximately 7% to 25% of the 760,000 ESKD patients in the United States [1–3]. AF is associated with disorganized atrial activation [4], increasing the risk of stroke, with ESKD patients experiencing a threefold higher annual incidence compared to those without AF [5]. Stroke in ESKD patients carries a poor prognosis, with one in three events being fatal within a year [5, 6]. While Vitamin K antagonists (VKAs) have traditionally been used for stroke prevention, their use in ESKD patients is challenging due to a heightened risk of bleeding [7, 8]. Newer direct oral anticoagulants like apixaban have been explored, yet retrospective studies indicate a higher relative risk of intracranial bleeding compared to no anticoagulation [8], leaving the decision to anticoagulate ESKD patients with non-valvular AF uncertain.

ESKD patients who undergo kidney transplantation experience a reduced risk of stroke but still face elevated mortality and cerebrovascular events compared to the general population [9, 10]. This risk escalates for transplant recipients with comorbid AF, correlating with poorer post-transplant outcomes [11, 12]. Studies show that ESKD patients with AF who receive kidney transplants experience a 37% higher risk of post-transplant stroke and a 2.4-fold increased mortality rate over five years compared to those without AF [11]. Moreover, nearly 7% of kidney transplant recipients encounter cerebrovascular events within three years post-transplant [11]. These findings underscore the importance of addressing AF in improving post-transplant outcomes.

Traditionally, VKAs have been prescribed for kidney transplant patients with AF [9], yet studies indicate suboptimal adherence to anticoagulation guidelines [13]. Similar to ESKD patients with AF, kidney transplant recipients with AF require vigilant anticoagulation management to mitigate stroke risk. While many studies have explored individuals with kidney transplants (KTx) or ESKD alongside comorbid AF and their risk of developing cerebral infarction or intracranial hemorrhage, these patient populations continue to face a heightened risk of stroke, encompassing both infarction and hemorrhage, compared to the general population [1, 4, 5, 7–10, 12–14]. Moreover, the dilemma of whether to administer anticoagulation remains a challenging aspect of medical decision-making for physicians.

This study aims to compare hospitalization rates for cerebral ischemic and hemorrhagic events in ESKD and KTx patients with and without AF using the National Inpatient Sample (NIS) from 2005–2009. Our objectives are to first compare baseline characteristics of ESKD with and without AF, followed by KTx patients with and without AF. Next, we compare hospitalization rates for ischemic and hemorrhagic cerebrovascular events in both ESKD and KTx populations with and without AF. Multivariable logistic regression analysis was then performed to compare the odds of each cerebral ischemic or hemorrhagic condition in patients with ESKD or KTx and AF compared to all ESKD hospitalizations included in this study, as well as demographic factors such as comorbidities, age, race, income, and patient location. We aim to demonstrate the impact of AF on hospitalization rates for ischemic and hemorrhagic cerebrovascular events in both ESKD and KTx patients.

## Materials and methods

### Data, data sources and sample details

The HCUP-NIS dataset underlies all planned analysis. Available through the Agency for Healthcare Research and Quality (AHRQ), the NIS is the largest publicly available all-payer inpatient healthcare database available to researchers. The dataset annually approximates a 20% stratified sample of all episode-level hospitalizations from U.S. community hospitals, and is primarily designed to produce U.S. regional and national estimates of inpatient utilization, access, cost, quality, and outcomes [15]. Available from 1988 to 2019, the complete dataset represents nearly 85% of all national hospitals over that time period. Each year includes around 7 million hospital visits, which represent nearly 35 million hospitalizations once correctly weighted [16]. IRB approval was not required since this study involved retrospective review of the National Inpatient Sample, a national, de-identified database. Patient consent was not obtained as it was not required given that all information was de-identified. The National Inpatient Sample was accessed on January 1, 2023 and the authors did not have access to information that could identify individual participants during or after data collection.

The utilized dataset consists of a 2005–2019 subset of the NIS database. Individuals were included in the analysis if they were hospitalized from January 1, 2005 through December 31, 2019. This analysis was performed at the patient level. First, we sorted through 113,032,831 discharges to match each performance measure to a set of corresponding ICD-9-CM from 2005 to 2014, or ICD-10-CM diagnosis codes from 2015 to 2019, summarized in S1 Table. Next, each ESKD patient in the dataset with at least one matching diagnosis code was compiled into a working dataset (*n* = 2,713,194). Additionally, all other listed diagnoses and procedures, discharge status, patient demographics, and charges associated to each discharge are included in the initial compiled dataset. Elixhauser comorbidity indices were then calculated for each recorded instance [17].

### Variables

**Fig 1:**
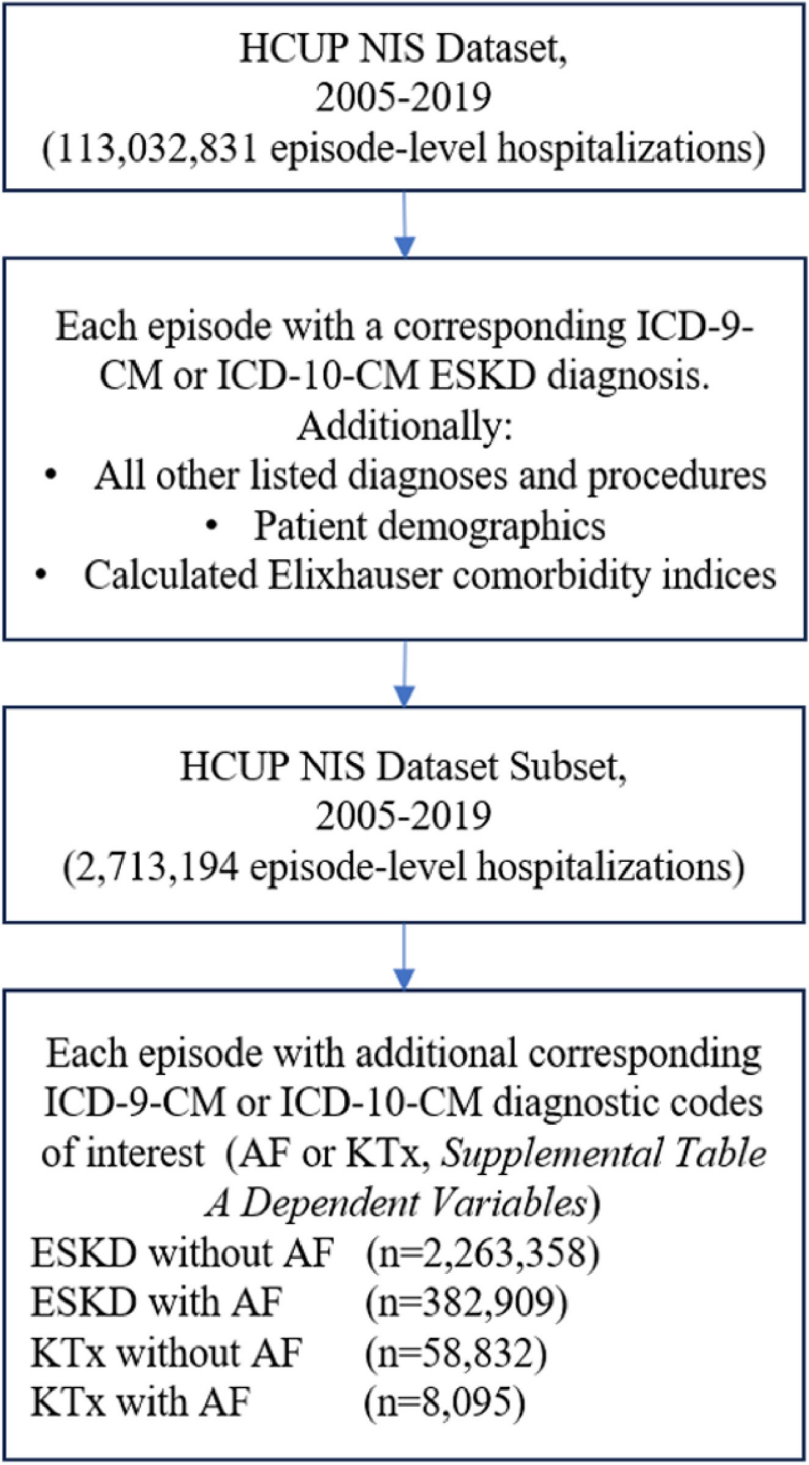
PRIMSA flow diagram representing the ESKD and KTx hospitalizations included in this study.

Fig 1 depicts a PRISMA flow diagram representing the ESKD hospitalizations included in this study. The inclusion criteria for this study focused on inpatient hospitalizations for end-stage kidney disease (ESKD) patients, with and without atrial fibrillation (AF), from 2005 to 2019. Additionally, it included patients with a history of ESKD and a kidney transplant (KTx), with and without AF, during the same period. Hospitalizations were included if patients had an ICD-9 or ICD-10 code for ESKD or KTx. The patients were categorized into two groups: the ESKD group, which included any hospitalization with an ESKD diagnosis and without KTx diagnosis code, and the KTx group, which included any hospitalization with both ESKD and KTx diagnoses codes. Patients with both ESKD and KTx were included in both groups, whereas those with only ESKD (no corresponding KTx diagnosis code) were included solely in the ESKD group, which allowed us to ascertain the impact of KTx. This is because it was presumed that patients with a history of kidney transplant were likely transplanted secondary to ESKD, thus no patients would only have diagnosis codes for only KTx without also having an ESKD diagnosis code. Exclusion criteria include hospitalization outside of these years, hospitalizations with incomplete records, and hospitalization records for patients less than 18 years old. S1 and S2 Tables include specific ICD9/10 codes utilized in this study, and more information regarding the specific IC9/10 inclusion/exclusion criteria can be found at www.ICD10data.com [18].

The independent variables included history of only ESKD, history of ESKD and AF, history of ESKD and KTx (hereafter referred to as “KTx”), or history of KTx and AF. Patients categorized as KTx includes only KTx patients without AF. Likewise, patients categorized as ESKD encompasses only those without AF.

Additionally, a number of demographic variables were included in the analysis. Age was expressed as the median age. Primary expected payer included Medicare, Medicaid, private insurance, self-pay, no charge, or other. Median household income was divided into quartiles ($1 - $28,999; $29,000 - $35,999; $36,000 - $46,999; > $47,000). Race was categorized into White, Black, Asian or Pacific Islander, Native American, or other, per the NIS guidelines. Patient location was categorized 6 groups per the National Center for Health Statistics guidelines ("Central" counties of metro areas of > 1 million population, "Fringe" counties of metro areas of >1 million population, counties in metro areas of 250,000–999,999 population, counties in metro areas of 50,000–249,999 population, micropolitan counties, not metropolitan or micropolitan counties).

The study outcomes include development of six key cerebral or ischemic conditions of interest, which were used as the dependent variables and are included in S2 Table. The key conditions of interest included five cerebral ischemic conditions and one cerebral hemorrhagic condition. The difference in the number of cerebral ischemic and hemorrhagic condition chosen was influenced by availability of data and accessibility of ICD9/10 codes. The five ischemic conditions included 1) Cerebral infarction due to thrombosis, embolism, occlusion, and stenosis (CITO), 2) Cerebral infarction due to thrombosis, embolism, and unspecified occlusion or stenosis of cerebral arteries (CIUO), 3) Artery occlusion and stenosis resulting in cerebral ischemia (AOSI), 4) Cerebral artery occlusion and stenosis resulting in cerebral ischemia (CAIO), 5) Intraoperative and postprocedural cerebrovascular infarction (IPI). The hemorrhagic conditions included 1) Nontraumatic Intracranial Hemorrhage (NIH). Each of these key cerebral or ischemic conditions of interest corresponds with a set of ICD-10-CM or corresponding ICD-9-CM diagnosis codes, summarized in S1 Table. ICD-9 diagnostic codes were used before October 1, 2015, and ICD-10 codes were used after this date. Each encounter with a matching diagnosis code was compiled into our dataset (n = 2,713,194).

### Analysis

A retrospective descriptive analysis was completed to investigate the probability difference of each cerebral ischemic or hemorrhagic condition of interest amongst ESKD patients without AF compared to those with AF. The same analysis was performed for KTx patients without AF compared to those with AF. Descriptive analysis was done using a t-test for continuous variables and Chi-squared/Fishers exact tests for categorical variables, depending on the sample size and the distribution of the variables included. Covariates included the Elixhauser comorbidity index and a series of sociodemographic proxies (i.e., age, race, primary expected payer, median household income, location), with complete variable details reported in S2 Table [17].

Next, multivariable logistic regression analysis was conducted using each cerebral or ischemic condition of interest as the dependent variable and ESKD or KTx and AF as the independent variables. This analysis aimed to determine the odds of hospitalization for each cerebrovascular condition in patients with ESKD or KTx and AF compared to all hospitalizations with a diagnosis of ESKD (Fig 1, n = 2,713,194). Additionally, multivariable logistic regression analysis included adjusting for the interactions of specific demographic factors previously mentioned to limit confounding. A two-sided significance level of < 0.05 was utilized to evaluate statistical significance for both the descriptive analysis and the regressions conducted in this study.

## Results

### Baseline and comparative characteristics for ESKD patients with and without AF

In Table 1, we present the baseline and comparative characteristics of ESKD patients hospitalized with AF. Out of 2,263,358 ESKD hospitalizations, 382,909 cases had concurrent AF. Patients with ESKD and AF were significantly older (mean age: 70.49 vs. 60.04 years, p < 0.001) and exhibited a higher burden of comorbidities (mean Elixhauser Comorbidity Index: 6.48 vs. 5.15, p < 0.001) compared to ESKD patients without AF. Demographic and socioeconomic disparities were noted, as ESKD patients with AF demonstrated a higher frequency of being White (58.87% vs. 38.92%, p < 0.001), covered by Medicare (85.21% vs. 72.55%, p < 0.001), and a lower frequency of belonging to the lowest income quartile ($1–28,999) (31.40% vs. 40.04%, p < 0.001).

**Table 1:** Baseline demographic characteristics for ESKD and KTx with and without AF.

	ESKD without AF	ESKD with AF	P-value	KTx without AF	KTx with AF	P-value
	(n = 2,263,358)	(n = 382,909)		(n = 58,832)	(n = 8,095)	
**Specification Controls**	** **	** **	** **	** **	** **	** **
Elixhauser Comorbidity Index (1–18), mean (sd)	5.15 (1.94)	6.48 (1.90)	<0.001	4.54 (1.89)	6.11 (1.92)	<0.001
Age, mean (sd)	60.04 (15.80)	70.49 (11.83)	<0.001	51.61 (15.66)	62.66 (11.29)	<0.001
Race, n (%)						
White	806,400 (38.92%)	206,492 (58.87%)	< 0.001	25,425 (47.94%)	4,423 (59.25%)	<0.001
Black	751,518 (36.27%)	82,436 (23.50%)		15,391 (29.02%)	1,666 (22.32%)	
Hispanic	352,371 (17.01%)	36,262 (10.34%)		8,172 (15.41%)	750 (10.05%)	
Asian or Pacific Islander	73,863 (3.57%)	14,842 (4.23%)		1,980 (3.73%)	358 (4.80%)	
Native American	24,658 (1.19%)	2,335 (0.67%)		486 (0.92%)	46 (0.62%)	
Other	62,929 (3.04%)	8,369 (2.39%)		1,580 (2.98%)	222 (2.97%)	
Primary expected payer, n (%)						
Medicare	1,639,464 (72.55%)	325,831 (85.21%)	< 0.001	42,688 (72.65%)	6,685 (82.64%)	<0.001
Medicaid	282,409 (12.50%)	19,241 (5.03%)		4,623 (7.87%)	270 (3.34%)	
Private Insurance	261,158 (11.56%)	30,482 (7.97%)		10,218 (17.39%)	1,009 (12.47%)	
Other	76,686 (3.39%)	6,817 (1.78%)		1,230 (2.09%)	125 (1.55%)	
Median household income national quartile for patient ZIP Code, n (%)						
$1-$28,999	885,564 (40.04%)	118,177 (31.40%)	< 0.001	18,443 (31.99%)	1,998 (25.12%)	<0.001
$29,000-$35,999	554,110 (25.05%)	95,144 (25.28%)		14,799 (25.67%)	2,022 (25.42%)	
$36,000-$46,999	452,814 (20.47%)	88,423 (23.49%)		13,509 (23.44%)	2,075 (26.09%)	
$47,000+	319,304 (14.44%)	74,612 (19.82%)		10,893 (18.90%)	1,859 (23.37%)	
Patient Location: NCHS Urban-Rural Code, n (%)						
Central counties of metro areas of > = 1 million population	888,323 (39.66%)	134,105 (35.43%)	< 0.001	20,581 (35.33%)	2,549 (32.01%)	<0.001
Fringe counties of metro areas of > = 1 million population	484,791 (21.65%)	94,576 (24.99%)		14,104 (24.21%)	2,206 (27.70%)	
Counties in metro areas of 250,000–999,999 population	398,218 (17.78%)	67,675 (17.80%)		10,947 (18.79%)	1,479 (18.57%)	
Counties in metro areas of 50,000–249,999 population	177,987 (7.95%)	31,338 (8.28%)		5,035 (8.64%)	668 (8.39%)	
Micropolitan counties	176,668 (7.89%)	30,784 (8.13%)		4,614 (7.92%)	620 (7.79%)	
Not metropolitan or micropolitan counties	113,678 (5.08%)	20,322 (5.37%)		2,966 (5.09%)	442 (5.55%)	

### Baseline and comparative characteristics for KTx patients with and without AF

Table 1 presents baseline and comparative characteristics for KTx patients with and without AF. Among 58,832 KTx hospitalizations, 8,095 cases had concurrent AF. Patients with KTx and AF were significantly older (mean age: 62.66 vs. 51.61 years, p < 0.001) and had a higher burden of comorbidities (mean Elixhauser Comorbidity Index: 6.11 vs. 4.54, p < 0.001). Demographic and socioeconomic differences were evident, as KTx patients with AF demonstrated a higher frequency of being White (59.25% vs. 4.94%, p < 0.001), covered by Medicare (82.64% vs. 72.65%), and a lower frequency of belonging to the lowest income quartile ($1–28,999) (25.12% vs. 31.99%, p < 0.001).

### Cerebral ischemic and hemorrhagic events in ESKD patients with and without AF

Among ESKD patients, those with AF exhibited a higher hospitalization rate for five of the six ischemic cerebrovascular conditions studied: CITO (0.11% vs. 0.08%, p < 0.001), CIUO (1.93% vs. 1.51%, p < 0.001), AOSI (1.37% vs. 0.93%, p < 0.001), and CAOI (0.48% vs. 0.42%, p < 0.001), compared to ESKD patients without AF (Table 2). However, ESKD patients with AF showed a lower hospitalization rate for IPI compared to ESKD without AF (0.88% vs. 0.97%, p < 0.001). ESKD patients with AF on the other hand demonstrated no differences in the odds of IPI when compared to the entire study population (OR = 0.973, 95% CI 0.799–1.187, p = 0.79). Notably, ESKD patients with AF had a lower hospitalization rate for the hemorrhagic condition of NIH (0.60% vs. 0.64%, p < 0.001) compared to ESKD patients without AF. In addition, ESKD patients with AF were found to be significantly less likely to be hospitalized with NIH compared to all ESKD patients, by nearly 17 percentage points across our sample (Table 4, OR = 0.835, 95% CI 0.795–0.877, p<0.001)

**Table 2:** Compares hospitalization rates for cerebral ischemic and hemorrhagic conditions in ESKD patients with and without AF.

	ESKD without AF	ESKD with AF	P-value
	(n = 2,263,358)	(n = 382,909)	
Cerebral Ischemic Conditions			
CITO	1,840 (0.08%)	426 (0.11%)	< 0.001
CIUO	34,175 (1.51%)	7,379 (1.93%)	< 0.001
AOSI	21,031 (0.93%)	5,263 (1.37%)	< 0.001
CAOI	9,581 (0.42%)	1,826 (0.48%)	< 0.001
IPI	21,990 (0.97%)	3,361 (0.88%)	<0.001
**Cerebral Hemorrhagic Condition**			
NIH	14,380 (0.64%)	2,301 (0.60%)	0.013

*Fisher’s Exact Test

### Cerebral ischemic and hemorrhagic events in KTx patients with and without AF

KTx patients with AF exhibited a higher hospitalization rate for CIUO (1.62% vs. 1.11%, p < 0.001) and AOSI (0.84% vs. 0.52%, p < 0.001) compared to KTx patients without AF (Table 3). However, they did not show significant differences in hospitalization rates for other ischemic conditions, including CITO, CAOI, or IPI, compared to KTx patients without AF (Table 3). Notably, KTx patients with AF exhibited a higher hospitalization rate for NIH (0.79% vs. 0.56%, p < 0.001) than KTx patients without AF. In addition, patients with KTx and AF were 1.504 times more likely to be hospitalized with NIH than all ESKD patients included in the analysis (Table 4, OR 1.504, 95% CI 1.134–1.996, p = 0.005). However, KTx patients with AF displayed no differences in the odds of being hospitalized with any of the cerebral ischemic conditions of interest studied, including IPI, compared to all ESKD hospitalizations included in this study (Table 4, OR = 0.961, 95% CI 0.21–4.489, p = 0.959).

**Table 3:** Compares hospitalization rates for cerebral ischemic and hemorrhagic conditions in KTx patients with and without AF.

	KiTx without AF	KiTx with AF	P-value
	(n = 58,832)	(n = 8,095)	
Cerebral Ischemic Conditions			
CITO	30 (0.05%)	7 (0.09%)	0.204*
CIUO	652 (1.11%)	131 (1.62%)	<0.001
AOSI	303 (0.52%)	68 (0.84%)	<0.001
CAOI	208 (0.35%)	28 (0.35%)	0.913
IPI	536 (0.91%)	75 (0.93%)	0.891
**Cerebral Hemorrhagic Condition**			
NIH	331 (0.56%)	64 (0.79%)	0.012

*Fisher’s Exact Test

**Table 4:** Multivariable regressions depicting the odds of each cerebral ischemic and hemorrhagic condition key conditions, race, primary expected payer, median household income, and patient location.

	Cerebral infarction due to thrombosis, embolism, occlusion, and stenosis (CITO)		Cerebral infarction due to thrombosis, embolism, and unspecified occlusion or stenosis of cerebral arteries (CIUO)		Artery Occlusion and stenosis resulting in cerebral ischemia (AOSI)		Cerebral artery occlusion and stenosis resulting in cerebral ischemia (CAOI)		Intraoperative and postprocedural cerebrovascular infarction (IPI)		Nontraumatic intracranial hemorrhages (NIH)	
	OR (95% CI)	p-value	OR (95% CI)	p-value	OR (95% CI)	p-value	OR (95% CI)	p-value	OR (95% CI)	p-value	OR (95% CI)	p-value
Ktx**5**	0.792 (0.537–1.17)	0.242	0.938 (0.864–1.019)	0.129	0.762 (0.676–0.86)	<0.001	1 (0.861–1.162)	0.999	0.809 (0.444–1.475)	0.49	0.981 (0.872–1.103)	0.748
ESKD and AF**6**	0.894 (0.795–1.006)	0.062	0.945 (0.919–0.973)	<0.001	0.875 (0.846–0.905)	<0.001	0.962 (0.908–1.019)	0.184	0.973 (0.799–1.187)	0.79	0.835 (0.795–0.877)	<0.001
KTx and AF**7**	1.172 (0.479–2.871)	0.728	1.112 (0.911–1.357)	0.296	1.045 (0.786–1.389)	0.762	0.987 (0.655–1.486)	0.949	0.961 (0.21–4.389)	0.959	1.504 (1.134–1.996)	0.005
Elixhauser Comorbidity Index (1–18)^1^	1.155 (1.132–1.177)	<0.001	1.122 (1.117–1.127)	<0.001	1.086 (1.079–1.092)	<0.001	0.914 (0.905–0.924)	<0.001	1.107 (1.07–1.145)	<0.001	1.087 (1.078–1.095)	<0.001
Age^1^	1.024 (1.02–1.027)	<0.001	1.02 (1.019–1.021)	<0.001	1.039 (1.038–1.04)	<0.001	1.029 (1.027–1.03)	<0.001	1.016 (1.011–1.02)	<0.001	1.006 (1.005–1.007)	<0.001
***Race***												
Black^2^	0.878 (0.786–0.981)	0.021	1.248 (1.217–1.28)	<0.001	0.592 (0.572–0.613)	<0.001	1.17 (1.114–1.229)	<0.001	0.881 (0.736–1.055)	0.168	1.209 (1.16–1.26)	<0.001
Hispanic^2^	0.855 (0.744–0.983)	0.028	1.053 (1.018–1.088)	0.002	0.741 (0.711–0.773)	<0.001	1.018 (0.956–1.084)	0.583	0.84 (0.661–1.068)	0.155	1.276 (1.214–1.34)	<0.001
Asian or Pacific Islander^2^	0.829 (0.653–1.053)	0.124	1.161 (1.1–1.225)	<0.001	0.787 (0.735–0.843)	<0.001	0.799 (0.712–0.897)	<0.001	1.009 (0.698–1.457)	0.963	1.973 (1.84–2.116)	<0.001
Native American^2^	0.493 (0.264–0.922)	0.027	0.864 (0.768–0.971)	0.015	0.925 (0.813–1.052)	0.233	0.894 (0.713–1.122)	0.335	0.426 (0.136–1.329)	0.141	1.171 (0.992–1.381)	0.062
Other^2^	0.858 (0.649–1.134)	0.282	1.092 (1.025–1.164)	0.007	0.809 (0.745–0.877)	<0.001	0.964 (0.849–1.093)	0.566	1.165 (0.781–1.738)	0.455	1.513 (1.386–1.651)	<0.001
***Primary Expected Payer***	*** ***	*** ***	*** ***	*** ***	*** ***	*** ***	*** ***	*** ***	*** ***	*** ***	*** ***	*** ***
Medicaid^3^	0.73 (0.603–0.884)	<0.001	0.981 (0.944–1.02)	0.34	0.666 (0.624–0.71)	<0.001	0.751 (0.691–0.817)	<0.001	0.677 (0.495–0.927)	0.015	1.029 (0.975–1.087)	0.301
Private Insurance^3^	1.092 (0.839–1.082)	0.24	1.18 (1.141–1.221)	<0.001	0.979 (0.935–1.026)	0.371	0.996 (0.931–1.066)	0.907	1.456 (1.178–1.799)	0.001	1.204 (1.144–1.266)	<0.001
Other^3^	0.977 (0.935–1.236)	0.87	1.092 (1.018–1.154)	0.007	0.658 (0.594–0.73)	<0.001	0.655 (0.564–0.76)	<0.001	0.878 (0.54–1.427)	0.599	1.281 (1.174–1.397)	<0.001
***Median Household Income National Quartile for Patient Zip Code***	*** ***	*** ***	*** ***	*** ***	*** ***	*** ***	*** ***	*** ***	*** ***	*** ***	*** ***	*** ***
$29,000-$35,999^4^	1.032 (0.92–1.157)	0.591	1.022 (0.995–1.05)	0.112	1.08 (1.043–1.118)	<0.001	1.018 (0.966–1.073)	0.509	1.059 (0.873–1.286)	0.559	1.014 (0.972–1.058)	0.51
$36,000-$46,999^4^	0.952 (0.839–1.082)	0.453	1.029 (0.999–1.059)	0.063	1.095 (1.055–1.136)	<0.001	1.069 (1.011–1.131)	0.02	0.991 (0.801–1.227)	0.936	1.006 (0.96–1.053)	0.81
$47,000+^4^	1.075 (0.935–1.236)	0.312	0.993 (0.96–1.028)	0.702	1.095 (1.051–1.142)	<0.001	0.988 (0.926–1.054)	0.717	1.097 (0.87–1.384)	0.435	1.064 (1.01–1.121)	0.2
***Patient Location***												
Fringe counties of metro areas of > = 1 million population^5^	0.894 (0.793–1.007)	0.065	1.037 (1.008–1.067)	0.001	1.085 (1.047–1.124)	<0.001	1.055 (1.001–1.112)	0.045	1.15 (0.951–1.39)	0.15	0.922 (0.882–0.964)	<0.001
Counties in metro areas of 250,000–999,999 population5	0.964 (0.849–1.094)	0.566	1.066 (1.034–1.098)	<0.001	1.188 (1.144–1.233)	<0.001	0.944 (0.89–1.001)	0.052	1.084 (0.88–1.336)	0.448	0.999 (0.955–1.046)	0.975
Counties in metro areas of 50,000–249,999 population^5^	0.843 (0.703–1.011)	0.066	1.114 (1.07–1.16)	<0.001	1.191 (1.133–1.252)	<0.001	1 (0.924–1.082)	0.998	0.812 (0.592–1.113)	0.194	0.924 (0.865–0.987)	0.02
Micropolitan counties^5^	1.001 (0.84–1.194)	0.991	1.148 (1.102–1.197)	<0.001	1.16 (1.102–1.221)	<0.001	1.003 (0.925–1.087)	0.945	0.795 (0.572–1.105)	0.172	0.913 (0.852–0.979)	0.01
Not metropolitan or micropolitan counties^5^	1.06 (0.865–1.299)	0.575	1.19 (1.133–1.25)	<0.0001	1.337 (1.261–1.417)	<0.001	0.937 (0.849–1.035)	0.201	1.104 (0.782–1.559)	0.572	0.945 (0.87–1.026)	0.179

^1^Reference group: ESKD

^2^Reference group: White

^3^ Reference group: Medicare

^4^ Reference group: Median household income national quartile for patient ZIP Code ($1–28,999)

^5^Reference Group: Patient location: central counties of metro areas of > = 1 million population

## Discussion

In this study, we utilized the NIS database to investigate hospitalization rates for ischemic and hemorrhagic cerebrovascular diseases in patients with ESKD or KTx and comorbid AF. Here, we present several observations that offer insights into the hospitalization rates for these cerebrovascular diseases in this specific patient population.

First, we observed that hospitalizations for five of the six studied cerebral ischemic events in ESKD, which encompassed CITO, CIUO, AOSI, and CAOI, were significantly more prevalent in those with comorbid AF when compared to ESKD patients without AF. Concurrently, hospitalizations for NIH in ESKD patients were significantly less common among those with AF compared to ESKD patients without AF. They also had lower odds of NIH compared to all ESKD hospitalization included in this study.

While this finding is firmly established in the literature [1, 5, 6, 8, 9, 12, 14], it remains evident that patients with both ESKD and AF continue to face an elevated risk of ischemic events when compared to those with ESKD alone, despite current therapeutic strategies. Although some of the ischemic events may be attributed to non-cardioembolic, atherosclerotic plaque rupture, their occurrence appears relatively similar to ESKD patients without AF. For those with ESKD and AF, a significant proportion of patients fall into categories: 1) not receiving appropriate therapy due to concerns about hemorrhagic risk, 2) experiencing therapy failure due to inherent limitations in their medication regimen (e.g., Direct-oral anticoagulants (DOACs) or VKAs), or 3) encountering therapy failure due to hemorrhagic complications (including cerebral events). The increased hospitalization rates for ischemic cerebrovascular disease in ESKD patients with AF emphasize the need for improved therapeutic management in this patient population.

Second, we found that KTx patients with AF experienced increased hospitalization rates for two of the six studied cerebrovascular ischemic conditions when compared to KTx patients without AF. In addition, KTx patients with AF demonstrated no differences in the odds of experiencing any of the ischemic conditions compared to all ESKD hospitalization in this study. However, KTx patients with AF did experience increased hospitalization rates for NIH compared to those without AF. In addition, KTx patients with AF demonstrated much higher odds of being hospitalized for NIH compared to the entire study population. It is known that KTx reduces the risk for ischemic stroke for patients with ESKD [9], but KTx recipients are still at increased risk for stroke compared to the general population [11]. In addition, prior literature has established that all types of strokes increase the mortality risk among KTx recipients [9]. Of particular note, hemorrhagic events have been reported as the most significant predictor of mortality [9]. Furthermore, additional studies have demonstrated that following kidney transplantation, AF is linked to the time it takes for an ischemic stroke to occur, while warfarin use has not shown a significant association with the risk of ischemic stroke [19]. Consequently, it is prudent to investigate alternative approaches beyond anticoagulation for the management of AF in KTx patients with the aim of diminishing the occurrence of hemorrhagic cerebral events.

Thirdly, no difference in hospitalization rates or odds for IPI was observed in either ESKD or KTx with AF compared to those without AF or to the entire study population. This finding may have implications for patients with ESKD and AF who are candidates for renal transplantation. It is known that there is potential for cerebral infarction due to heightened coagulation during the early postoperative period which stems from systemic inflammation [20]. At the same time, anticoagulation is relatively contraindicated during the perioperative period [21]. For patients with AF, current guidelines suggest discontinuing warfarin within 5 days and DOACs within 2 days of a high-risk operation [21]. Previous studies investigating perioperative ischemic stroke risk in kidney transplant recipients, both with and without AF, have indicated a rate of 1.3% with 39.7% of these events occurring either during the hospital stay or in the immediate perioperative period [22]. In summary, the absence of significant differences in hospitalization rates for IPI among ESKD and KTx patients with and without AF raises important considerations for the perioperative management of this patient population.

These findings highlight distinct patterns: ESKD patients with AF exhibited higher hospitalization rates for ischemic stroke compared to those without AF, whereas KTx patients with AF showed increased hospitalizations for hemorrhagic stroke compared to their counterparts without AF. Despite this, both patient groups remain at elevated risk of ischemic stroke compared to the general population [11]. While not explored in this study, it’s worth noting that Left Atrial Appendage Occlusion (LAAO) emerges as a potentially underappreciated therapeutic option for this patient population. Several observational studies have reported no significant differences in mortality or periprocedural cerebrovascular events between patients with ESKD and matched cohorts without ESKD who have received the device [7, 23, 24]. In addition, the Left Atrial Appendage Occlusion with WATCHMAN Device in Patients with Non-valvular Atrial Fibrillation and ESKD on Hemodialysis (WATCH-HD) study is an ongoing observational prospective registry aimed at enrolling participants undergoing dialysis. Expected to conclude this year, this study is poised to become the largest investigation of LAAO in dialysis patients to date [7, 25].

## Limitations

Limitations of this study include its retrospective design which introduces inherent limitations, which include selection bias, data accuracy and coding which depends on the coding practices and completeness of the information recorded in the NIS, generalizability to populations outside the United States, and a lack of cause-effect relationship due to the observational nature of the study. Additionally, some limitations of the database include unmeasured confounders, lack of clinical details that the NIS does not provide, management variability, potential confounding despite statistical adjustments, and lack of long-term outcomes such as mortality or functional outcomes. Further, inclusion of patients who received ESKD and KTx in both groups may limit generalizability of the results, however, we were unable to form discrete groupings due to inherent limitations with the dataset. Despite these limitations, this analysis provides insights into the rates of hospitalization for cerebrovascular diseases in patients with ESKD or KTx with and without AF.

## Conclusion

In conclusion, our findings reveal distinct patterns in hospitalization rates between ESKD and KTx patients with AF compared to those without AF, with ESKD patients with AF exhibiting higher rates of ischemic stroke compared to ESKD patients without AF and KTx patients with AF showing increased hospitalizations for hemorrhagic stroke compared to those without AF. Our study demonstrated no differences in IPI for ESKD or KTx patients with AF compared to those without AF. Together, these findings demonstrate the impact of AF on hospitalization rates for ischemic and hemorrhagic cerebrovascular events in both ESKD and KTx patients.

## Supporting information

S1 Table ICD-9 and 10 codes for each diagnosis were utilized in this study. (DOCX)

S2 Table Descriptions of each variable utilized in this study. (DOCX)

S1 Graphical abstract (PPTX)
