# Supplementary material for: Comparative analysis of ischemic and hemorrhagic stroke hospitalization rates in end-stage kidney disease and kidney transplant patients with and without atrial fibrillation
Source: PLoS One. 2024 Dec 16;19(12):e0310181. doi: 10.1371/journal.pone.0310181 (PMC11649131; doi:10.1371/journal.pone.0310181)
Supplement: S1 Table — (DOCX) [file pone.0310181.s001.docx]

| **Supplemental Table 1: ICD-10 and ICD-9 Descriptions** |  |  |
| --- | --- | --- |
| **Diagnosis** | **ICD-10** | **ICD-9** |
| **End-Stage Kidney Disease** | N18.6 | 585.6 |
| **Atrial Fibrillation** |  | 427.31 |
|  | I48.0 I48.1 I48.11 I48.19 I48.2 I48.20 I48.21 |  |
|  |  |  |
|  |  |  |
| **Kidney Transplant** | Z94.0, Z48.22 | 556.9, V42.0 |
| **Cerebral infarction due to thrombosis, embolism, occlusion, and stenosis** | I63.0 I63.00 I63.01 I63.011 I63.012 I63.013 | 433.91 433.21 433.01 433.11 433.81 433.91 |
|  | I63.019 I63.02 I63.03 I63.031 I63.032 I63.033 I63.039 I63.09 I63.1 I63.10 I63.11 I63.111 I63.112 I63.113 I63.119 I63.12 I63.13 I63.131 I63.132 I63.133 I63.139 I63.19 I63.2 I63.20 I63.21 I63.211 I63.212 I63.213 I63.219 I63.23 I63.231 I63.232 I63.233 I63.239 I63.29 |  |
| **Cerebral infarction due to thrombosis, embolism, and unspecified occlusion or stenosis of cerebral arteries** | I63.3 I63.30 I63.31 I63.311 I63.312 | 434.01 434.11 434.91 |
|  | I63.313 I63.319 I63.32 I63.321 I63.322 I63.323 I63.329 I63.33 I63.331 I63.332 I63.333 I63.339 I63.34 I63.341 I63.342 I63.343 I63.349 I63.39 I63.6 I63.4 I63.40 I63.41 I63.411 I63.412 I63.413 I63.419 I63.42 I63.421 I63.422 I63.423 I63.429 I63.43 I63.431 I63.432 I63.433 I63.439 I63.44 I63.441 I63.442 I63.443 I63.449 I63.49 I63.5 I63.50 I63.51 I63.511 I63.512 I63.54 I63.541 I63.542 I63.543 I63.549 I63.59 I63.8 I63.81 I63.89 I639 |  |
| **Artery occlusion and stenosis resulting in cerebral ischemia** | I65.0 I65.01 I65.02 I65.03 I65.09 | 433.20 433.00 433.10 433.80 433.90 |
|  | I65.1 I65.2 I65.21 I65.22 I65.23 I65.29 I65.8 I65.9 |  |
| **Cerebral artery occlusion and stenosis resulting in cerebral ischemia** | I66.0 I66.01 I66.02 I66.03 I66.09 | 434.00 434.10 434.90 437.1 435.9 |
|  | I66.1 I66.11 I66.12 I66.13 I66.19 I66.2 I66.21 I66.22 I66.23 I66.29 I66.3 I66.8 I66.9 I67.8 I67.81 I67.82 I67.84 I67.848 |  |
| **Non-Traumatic Intracranial Hemorrhage** | I60.0 I60.00 I60.01 I60.02 I60.1 I60.10 | 430 431 432.1 432.0 432.9 |
|  | I60.11 I60.12 I60.2 I60.3 I60.30 I60.31 I60.32 I60.4 I60.5 I60.50 I60.51 I60.52 I60.6 I60.7 I60.8 I60.9 I61.0 I61.1 I61.2 I61.3 I61.4 I61.5 I61.6 I61.8 I61.9 I62.0 I62.00 I62.01 I62.02 I62.03 I62.1 I62.9 |  |
| **Intraoperative and postprocedural Infarction** | I97.8 I97.81 I97.811 I97.82 I97.821 | 997.02 |

**Supplemental Table 1.** ICD-9 and 10 codes for each diagnosis were utilized in this study.
