# Supplementary figures and images for: Comparative analysis of ischemic and hemorrhagic stroke hospitalization rates in end-stage kidney disease and kidney transplant patients with and without atrial fibrillation
Source: PLoS One. 2024 Dec 16;19(12):e0310181. doi: 10.1371/journal.pone.0310181 (PMC11649131; doi:10.1371/journal.pone.0310181)

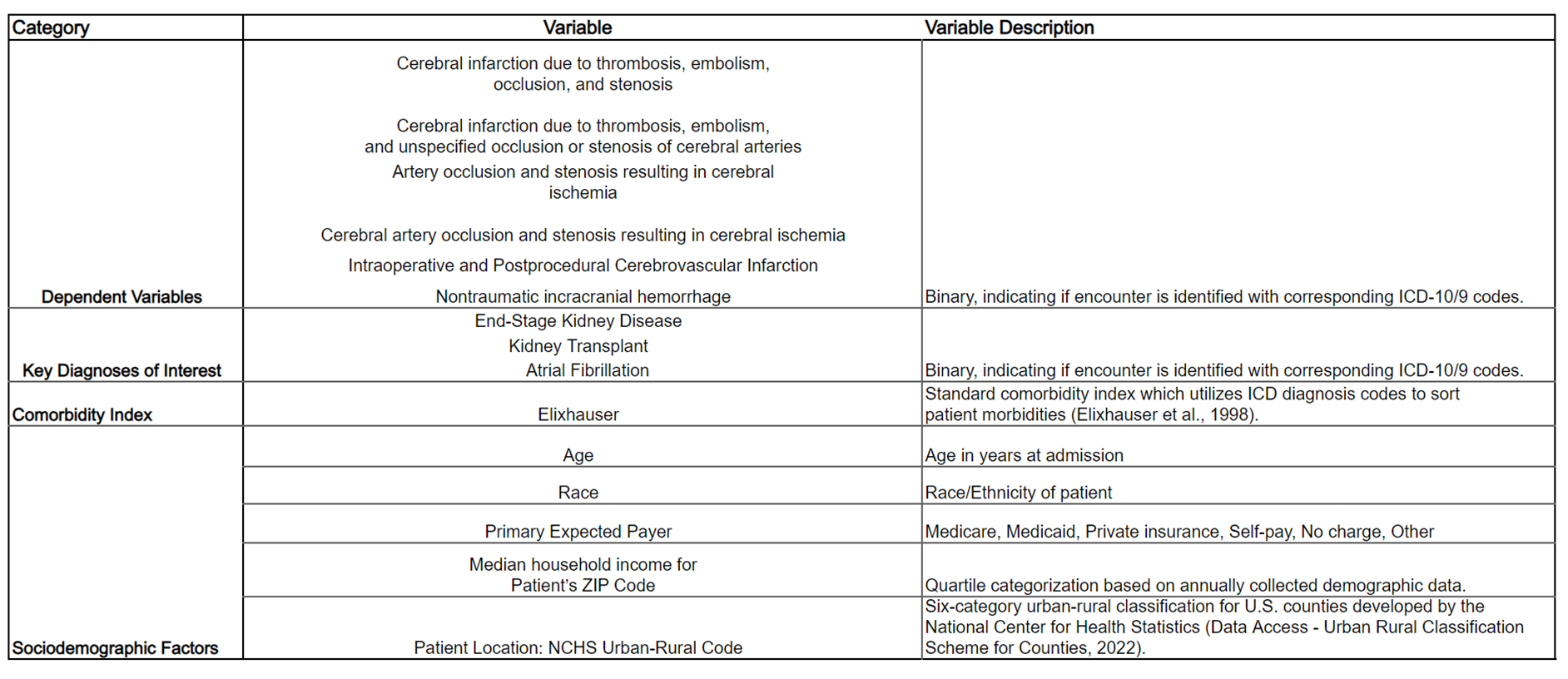


**Supplemental Table 2.** Descriptions of each variable utilized in this study.

Supplement: S2 Table — (DOCX) [file pone.0310181.s002.docx]
