# Supplementary material for: Comparative analysis of ischemic and hemorrhagic stroke hospitalization rates in end-stage kidney disease and kidney transplant patients with and without atrial fibrillation
Source: PLoS One. 2024 Dec 16;19(12):e0310181. doi: 10.1371/journal.pone.0310181 (PMC11649131; doi:10.1371/journal.pone.0310181)
Supplement: S1 Graphical abstract — (PPTX) [file pone.0310181.s003.pptx]

## Slide 1
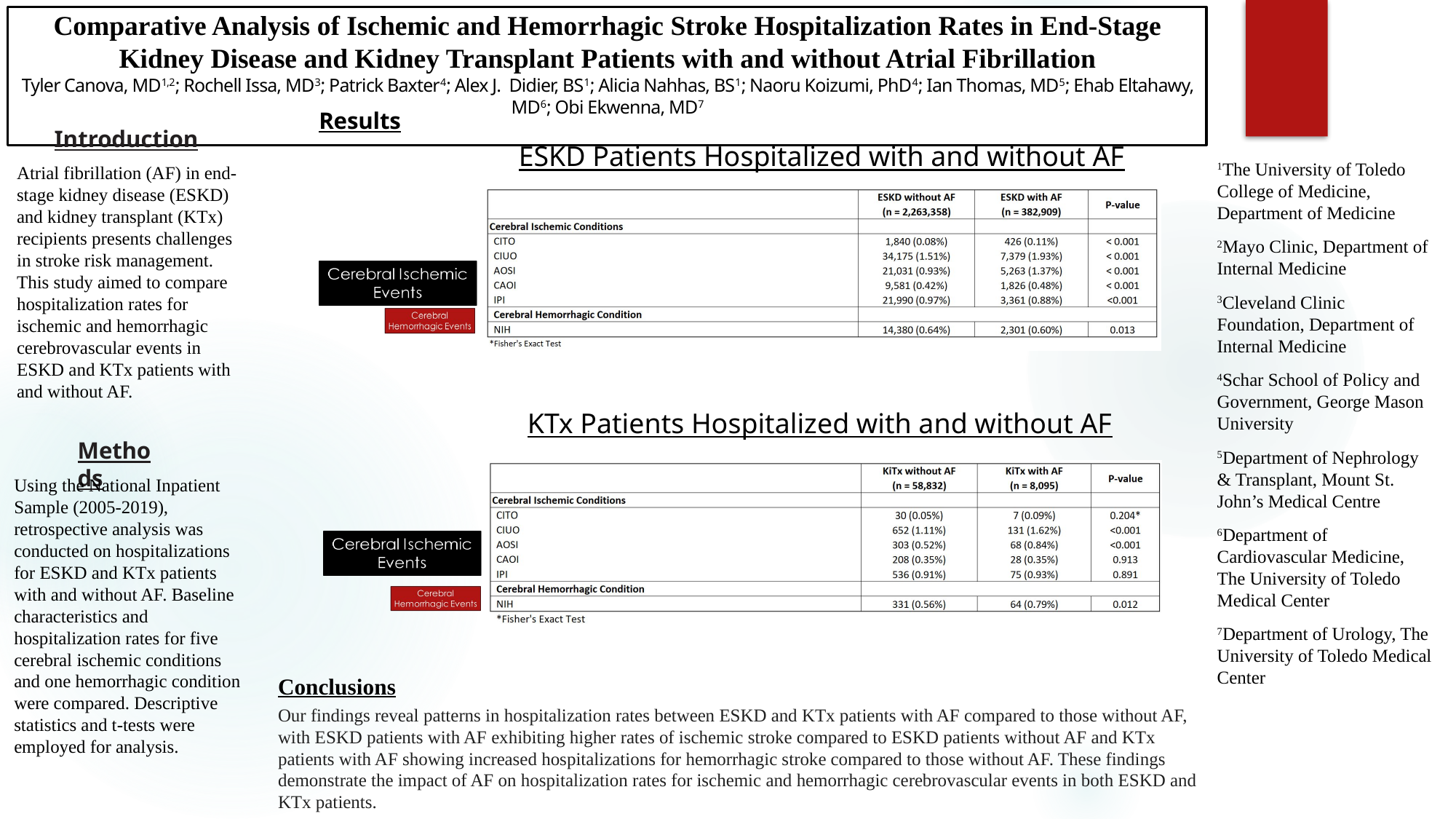

# Comparative Analysis of Ischemic and Hemorrhagic Stroke Hospitalization Rates in End-Stage Kidney Disease and Kidney Transplant Patients with and without Atrial Fibrillation Tyler Canova, MD1,2; Rochell Issa, MD3; Patrick Baxter4; Alex J. Didier, BS1; Alicia Nahhas, BS1; Naoru Koizumi, PhD4; Ian Thomas, MD5; Ehab Eltahawy, MD6; Obi Ekwenna, MD7
Results
Introduction
ESKD Patients Hospitalized with and without AF
1The University of Toledo College of Medicine, Department of Medicine
2Mayo Clinic, Department of Internal Medicine
3Cleveland Clinic Foundation, Department of Internal Medicine
4Schar School of Policy and Government, George Mason University
5Department of Nephrology & Transplant, Mount St. John’s Medical Centre
6Department of Cardiovascular Medicine, The University of Toledo Medical Center
7Department of Urology, The University of Toledo Medical Center
Atrial fibrillation (AF) in end-stage kidney disease (ESKD) and kidney transplant (KTx) recipients presents challenges in stroke risk management. This study aimed to compare hospitalization rates for ischemic and hemorrhagic cerebrovascular events in ESKD and KTx patients with and without AF.
KTx Patients Hospitalized with and without AF
Methods
Using the National Inpatient Sample (2005-2019), retrospective analysis was conducted on hospitalizations for ESKD and KTx patients with and without AF. Baseline characteristics and hospitalization rates for five cerebral ischemic conditions and one hemorrhagic condition were compared. Descriptive statistics and t-tests were employed for analysis.
Conclusions
Our findings reveal patterns in hospitalization rates between ESKD and KTx patients with AF compared to those without AF, with ESKD patients with AF exhibiting higher rates of ischemic stroke compared to ESKD patients without AF and KTx patients with AF showing increased hospitalizations for hemorrhagic stroke compared to those without AF. These findings demonstrate the impact of AF on hospitalization rates for ischemic and hemorrhagic cerebrovascular events in both ESKD and KTx patients.
Conclusion line 1
Conclusion line 2
Conclusion line 3
